# Impaired tumor immune response in metastatic tumors is a selective pressure for neutral evolution in CRC cases

**DOI:** 10.1371/journal.pgen.1009113

**Published:** 2021-01-21

**Authors:** Shotaro Sakimura, Satoshi Nagayama, Mitsuko Fukunaga, Qingjiang Hu, Akihiro Kitagawa, Yuta Kobayashi, Takanori Hasegawa, Miwa Noda, Yuta Kouyama, Dai Shimizu, Tomoko Saito, Atsushi Niida, Yusuke Tsuruda, Hajime Otsu, Yoshihiro Matsumoto, Hiroki Uchida, Takaaki Masuda, Keishi Sugimachi, Shin Sasaki, Kazutaka Yamada, Kazuki Takahashi, Hideki Innan, Yutaka Suzuki, Hiromi Nakamura, Yasushi Totoki, Shinichi Mizuno, Masanobu Ohshima, Tatsuhiro Shibata, Koshi Mimori

**Affiliations:** 1 Department of Surgery, Kyushu University Beppu Hospital, Beppu, Japan; 2 Department of Anesthesiology and Critical Care Medicine, Graduate School of Medical Sciences, Kyushu University, Fukuoka, Japan; 3 Gastroenterological Center, Department of Gastroenterological Surgery, Cancer Institute Hospital, Japanese Foundation for Cancer Research, Tokyo, Japan; 4 Division of Health Medical Data Science, Health Intelligence Center, Institute of Medical Science, University of Tokyo, Tokyo, Japan; 5 Laboratory of DNA Information Analysis, Human Genome Center, Institute of Medical Science, University of Tokyo, Tokyo, Japan; 6 Department of Surgery, Omori Red Cross Hospital, Tokyo, Japan; 7 Department of Surgery, Takano Hospital, Kumamoto, Japan; 8 Department of Evolutionary Studies of Biosystems, SOKENDAI, The Graduate University for Advanced Studies, Tokyo, Japan; 9 Medical Genome Sciences, Graduate School of Frontier Sciences, University of Tokyo, Chiba, Japan; 10 Division of Cancer Genomics, National Cancer Center Research Institute, Tokyo, Japan; 11 Center for Advanced Medical Innovation, Kyushu University, Fukuoka, Japan; 12 Division of Genetics, Cancer Research Institute, Kanazawa University, Kanazawa, Japan; 13 Laboratory of Molecular Medicine, Human Genome Center, Institute of Medical Science, University of Tokyo, Tokyo, Japan; Academic Medical Center, NETHERLANDS

## Abstract

A Darwinian evolutionary shift occurs early in the neutral evolution of advanced colorectal carcinoma (CRC), and copy number aberrations (CNA) are essential in the transition from adenoma to carcinoma. In light of this primary evolution, we investigated the evolutionary principles of the genome that foster postoperative recurrence of CRC. CNA and neoantigens (NAG) were compared between early primary tumors with recurrence (CRCR) and early primary tumors without recurrence (precancerous and early; PCRC). We compared CNA, single nucleotide variance (SNV), RNA sequences, and T-cell receptor (TCR) repertoire between 9 primary and 10 metastatic sites from 10 CRCR cases. We found that NAG in primary sites were fewer in CRCR than in PCRC, while the arm level CNA were significantly higher in primary sites in CRCR than in PCRC. Further, a comparison of genomic aberrations of primary and metastatic conditions revealed no significant differences in CNA. The driver mutations in recurrence were the trunk of the evolutionary phylogenic tree from primary sites to recurrence sites. Notably, *PD-1 a*nd *TIM3*, T cell exhaustion-related molecules of the tumor immune response, were abundantly expressed in metastatic sites compared to primary sites along with the increased number of CD8 expressing cells. The postoperative recurrence-free survival period was only significantly associated with the NAG levels and TCR repertoire diversity in metastatic sites. Therefore, CNA with diminished NAG and diverse TCR repertoire in pre-metastatic sites may determine postoperative recurrence of CRC.

## Author summary

We found that copy number aberrations (CNAs) may be the most important selective pressure promoting cancer evolution from early-to-advanced tumors in primary sites. The diminished neoantigens (NAG) in cancer cells and the diverse TCR repertoire in cytotoxic T cells were crucial for the onset of postoperative recurrence during genomic neutral evolution, along with clonal CNA and several driver SNVs from primary to recurrent sites. Therefore, cancer metastasis could be prevented by activating CTL at the premetastatic sites before priming of the metastatic process.

## Introduction

To improve the outcome and prognoses in colorectal cancer (CRC) patients, a deeper understanding of the evolutionary process is needed, such as the identification of the first evolutionary steps from precancerous to advanced tumors in primary sites and the second evolution from primary to recurrent sites. In the current study, we focused on the selective pressures affecting evolutionary processes that drive cancer recurrence, such as genomic aberrations and tumor immune response-related factors.

Genetic heterogeneity in tumor tissues drives cancer evolution and presumably causes therapeutic and diagnostic difficulties in precision medicine. Recent technological innovations, such as multi-regional analysis, enabled a deeper understanding of intra-tumor heterogeneity [1–6]. To study the evolution of advanced primary tumors, we previously implemented multiregional analysis of advanced CRC cases to elucidate the pathogenic mechanisms of this metastatic disease [7]. Advanced colorectal cancer harbors extensive intra-tumor heterogeneity (ITH) that is shaped by neutral evolution [7,8]. Additionally, we conducted multiregional whole-exome sequencing (WES) on 10 precancerous and early colorectal tumors and showed that early tumors accumulated a higher proportion of subclonal driver mutations, such as in *KRAS* and *APC*, than advanced tumors [8]. Therefore, we concluded that subclonal mutations are subject to a selective sweep of Darwinian natural selection evolution in early tumorigenesis [9], while neutral evolution is dominant in advanced tumors. In other words, the evolutionary principle underlying ITH shifts from Darwinian to neutral evolution during primary colorectal tumor progression [9]. Therefore, following our previous studies, we aimed to disclose the evolutionary style between primary and recurrence in CRC cases.

In our previous studies of CRC primary tumor evolution, it is worth restating that the number of arm-level copy number aberrations (CNA) increased exponentially from adenoma to carcinoma. Several studies identified critical genes along with CNA that are essential factors of recurrence; for example, Jamal-Hanjani et al. reported that intra-tumor heterogeneity mediated through CNA was associated with an increased risk of recurrence or death in small cell lung cancer [10]. Bakhoum et al. compared CNA between primary and recurrence tumors and reported that primary breast tumor had near-diploid (2n) karyotypes, while metastases were enriched for cells with near-triploid (3n) karyotypes [11]. However, Davoli et al. showed that the highly arm level CNA tumors had reduced expression of markers of cytotoxic infiltrating immune cells, especially CD8+ T cells, and increased expression of cell proliferation markers [12]. Therefore, in order to identify dominant factors that determine the susceptibility of tumors to recur or metastasize, we should focus not only on genomic aberrations, but also on tumor immune response-related genes whose expression is affected by arm-level CNA.

The current study included 10 cases of stage I or II CRC with postoperative recurrence. We analyzed and compared genomic aberrations, such as copy number aberrations (CNAs) and single nucleotide variants (SNVs) between primary tumors with postoperative recurrence (CRC with recurrence; CRCR) and those without recurrence for more than two years (Precancerous or early CRC; PCRC) to identify crucial selective pressures on primary tumors with postoperative recurrence. We constructed an evolutionary phylogenic tree of mutated driver genes between 9 primary sites and 10 recurrent/metastatic sites in 10 CRCR cases and compared clonality of mutated driver genes and CNA between primary and metastatic sites. We then compared neoantigens (NAG), T cell receptor (TCR) repertoires, and other tumor immune response-related molecules, including T cell exhaustion markers, between primary and metastatic sites to identify potential bona-fide factors that establish postoperative recurrence of CRC.

## Results

### Selective pressures in primary tumors for postoperative recurrence

#### Comparison of primary genomic aberrations between PCRC and CRCR

**Fig 1:**
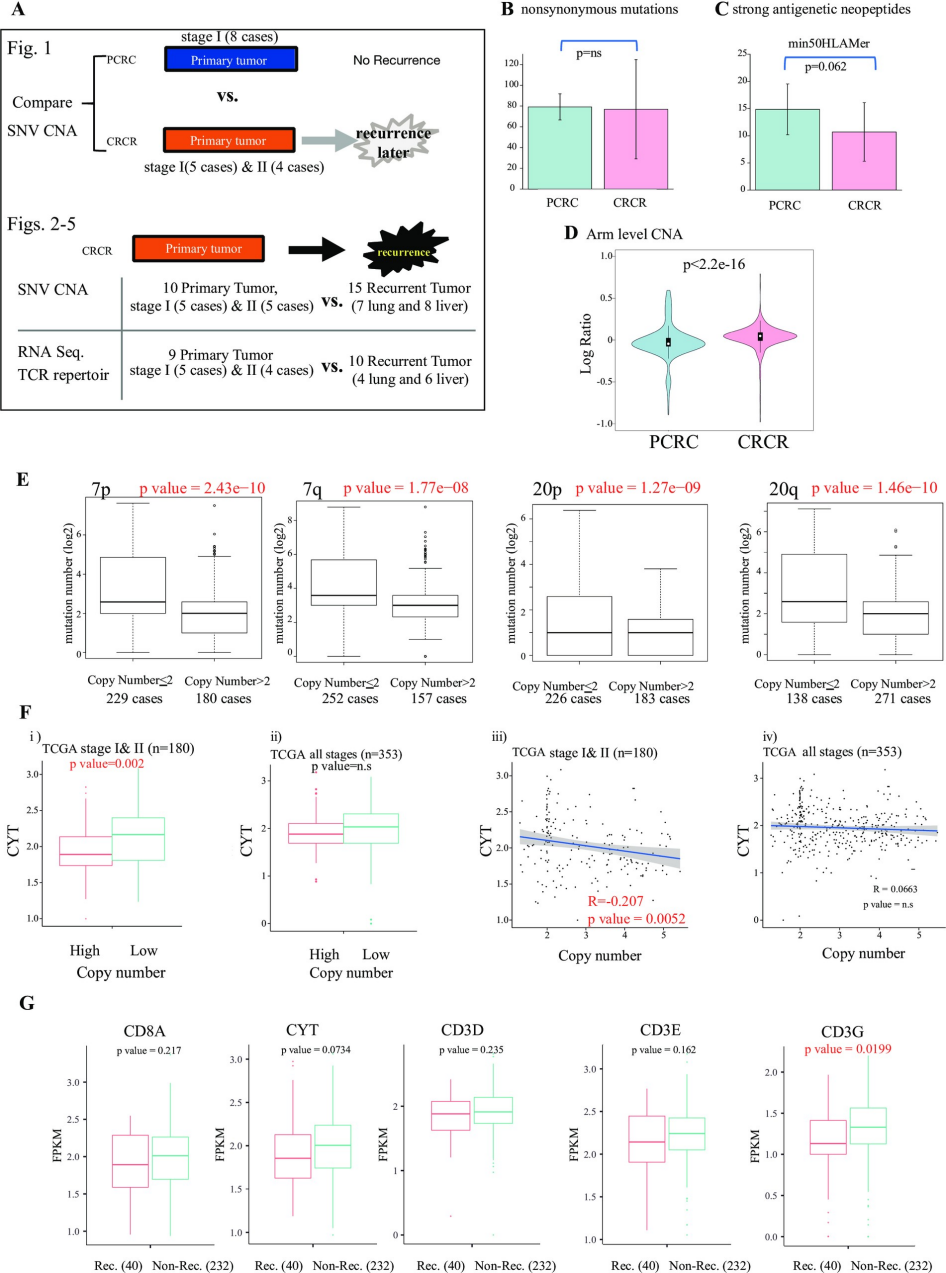
Neoantigens and copy number alterations (CNAs) in in-house cases and the TCGA database. **A)** Concept of the study design. Comparisons of the number of single nucleotide variants (SNVs), neoantigens (NAGs), and arm-level CNAs between eight primary tumors from early colorectal cancer cases without recurrence (PCRC) and eight primary tumors from CRC cases with recurrence (CRCR) postoperatively (Fig 1). In addition, we compared SNV, CNA, and RNA seq between primary and metastatic sites (Figs 2–5). **B)** Comparison of the average number of non-synonymous mutations between PCRC and CRCR. **C)** Neopeptides with a half-maximal inhibitory concentration (IC_50_) of less than 50 nM were classified as having strong antigenicity. Comparison of antigenicity between PCRC and CRCR. **D)** Comparison of arm-level copy numbers (CNs) between eight PCRCs and eight CRCRs. Log-ratios of total CNAs were compared between PCRC and CRCR (*p* < 2.2e-16). **E)** The number of mutations differed between chromosomal loci with CNs≤2 compared with those with CNs>2 at representative chromosome 7p, 7q, 20p, and 20q of all chromosome (**S2 Table**). **F)** The tumor immune response was evaluated by cytolytic activity (CYT). i) CYT levels in 180 cases of stage I and II CRC in TCGA; ii) CYT levels in all 353 cases; iii and iv) association between CYT and ploidy determined by scatter analysis. **G)** There were two groups: 40 cases of stage I/II CRC with postoperative recurrence from TCGA and 232 cases of stage I/II CRC without postoperative recurrence from TCGA. We demonstrated *CD8A*, *CD3G*, *CD3E*, *CD3G* expression and CYT in all cases (**S3 Table**).

Our study design is summarized in **Fig 1A**. We compared the total number of nonsynonymous mutations between eight PCRCs and eight CRCRs with respect to the number of SNVs (**Fig 1B**) and neoantigens (NAGs) (**Fig 1C**) in primary sites. We identified neopeptides and their binding affinities to patient major histocompatibility complex (MHC) molecules and defined neopeptides as having strong antigenicity with half-maximal inhibitory concentration (IC_50_) of less than 50 nM. The antigenicity tended to be stronger in PCRC than CRCR (**Fig 1C**).

Next, we compared the arm-level CNAs (ploidy) of our CRC cohort and found that CNAs were much more frequent in CRCRs than PCRCs (*p* < 2.2e-16) (**Fig 1D**). To verify the inverse association between the number of SNVs and arm-level CNAs, we evaluated 409 CRC cases in TCGA. Representative clonal amplification in CRC cases was observed on chromosomes 7p, 7q, 20p, and 20q (**Fig 1E**). We demonstrated an association between whole chromosomes in CRC cases from the TCGA database and found that 22 out of 44 arms (50%) showed a significant inverse association (q-value < 0.1) (**S2 Table**). This inverse association between aneuploidy and mutation burden seems feasible based on the former findings reported by Taylor et al [13].

#### Hampered tumor immune response in CRC with high CNA

We evaluated the tumor immune response of CRC cases and found that cytolytic activity (CYT) was significantly higher in CRCs with low CN than high CN **(Fig 1F, i, ii**). Moreover, a significant difference in CYT between high and low CN was observed only in stage I and II CRCs (*p* < 0.01). We also examined the association between CN and CYT and found that many diploid CRC cases (ploidy = 2.0) had the highest CYT value in comparison to CRC cases with multi-ploidy (**Fig 1F, iii, iv**). CYT and CN levels were inversely associated (*p* = 0.0052, R = -0.207) only in stage I and II CRCs. Therefore, the associations between cytolytic activity of CTL and antigenicity induced by SNVs on diploid chromosomes in early CRC cases were maintained in a logical manner.

#### Tumor immune response is significantly disrupted in CRCR compared to PCRC

CYT was lower in recurrent-positive (Recurrent; n = 40) cases than in recurrent-negative (Non-Recurrent; n = 232) cases in TCGA (**Fig 1G**). In addition to CYT, we compared the expression of tumor immune response-related genes and found that expression of *CD3G* gene was significantly higher in 232 Non-Recurrent cases (*p* = 0.0199) compared to 40 Recurrent cases with statistical significance (**Fig 1G** and **S3 Table**).

#### Clinical significance of cytolytic activity (CYT) associations

We applied CYT as a key indicator for evaluating tumor immune response in CRC cases. There was no significant association between CYT and OS in 272 cases of stage I and II colorectal cancer (CRC); however, 154 cases of CRC with higher CYT showed a significantly better prognosis in relapse free survival (RFS) than 118 cases with lower CYT (*p* < 0.05) (**S1 Fig**). Therefore, in primary tumors, CNA is the most critical and necessary conditions for fostering postoperative recurrence in CRC cases.

Based on the comparative data of genomic aberrations (SNV and CNA) in post-operative primary tumors with recurrence and without recurrence, we hypothesized that the most definitive selective pressure for recurrence may be copy number aberrations, which are significantly associated with diminished neoantigens due to the lower frequency of somatic mutations in primary tumors. Therefore, in further experiments, we compared the CNA between primary tumors and recurrent tumors in 10 CRCR cases. Initially, to identify the metastasis specific driver genes, we constructed phylogenic evolutional trees of driver mutations from primary sites to metastatic sites.

### Evolution from primary to recurrent cancers

#### Comparison of genomic aberrations in primary and recurrent cancers

**Fig 2:**
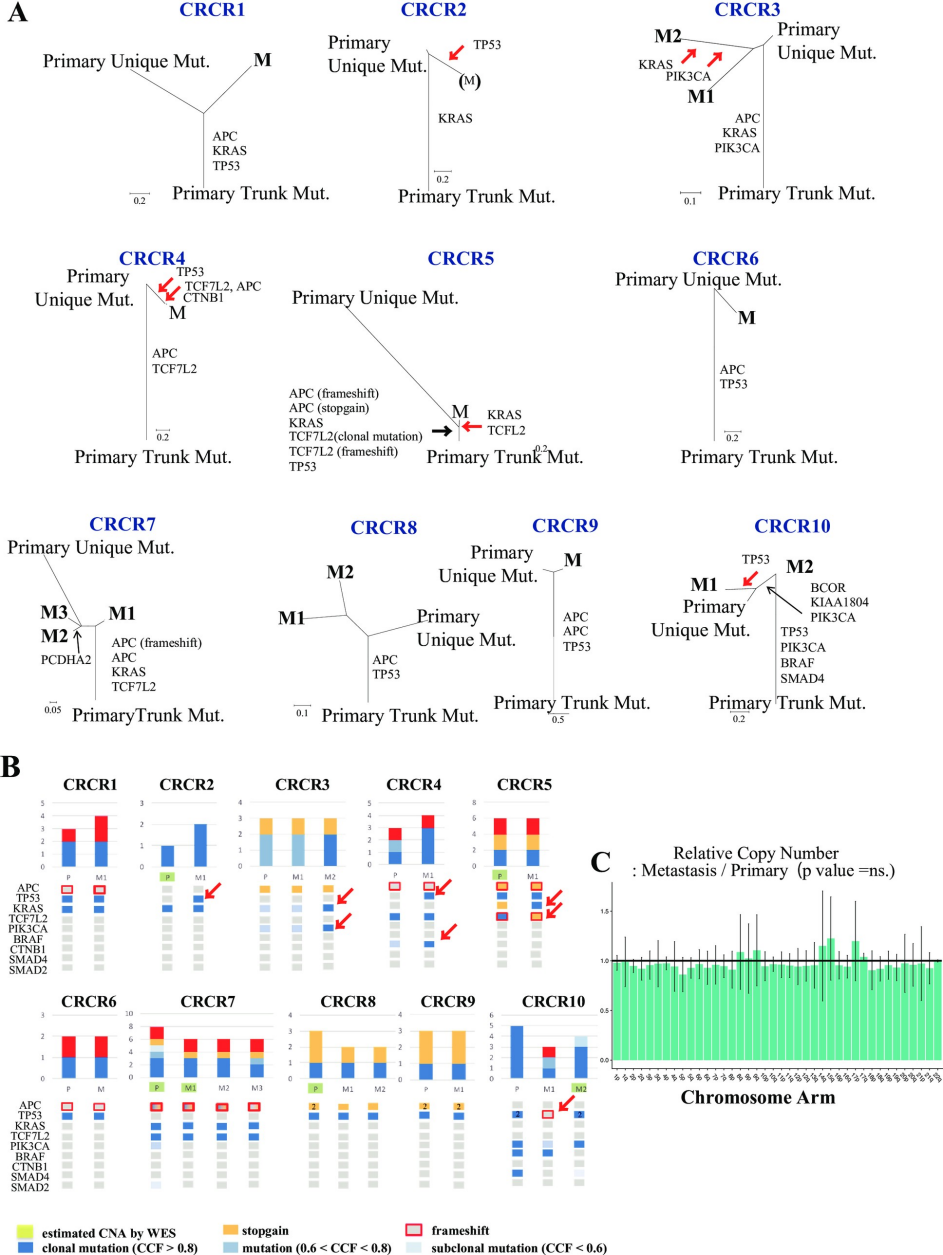
Phylogenic tree of the evolution from primary to recurrent sites. **A)** Primary Unique Mut: single nucleotide variants (SNVs) were observed in primary tissues but not in metastatic sites. Primary Trunk Mut: SNVs in both primary tumors and metastatic tumors. M: SNVs in metastatic recurrent cancers only. The list of consensus driver genes is shown. Most driver mutations were observed from primary to metastatic sites; although, CRCR2 (M) was excluded from further analysis because the sample was exposed to multiple chemotherapies for 150 d after the diagnosis of recurrent sites. However, another 10 recurrent tumors were removed within 30 d without any treatment. **B)** Comparison of the clonality of mutated driver genes between primary and metastatic sites. Most mutated driver genes in primary sites were identical to metastatic sites (neutral evolution). However, a few subclonal mutated driver genes (red arrows) were observed in metastatic sites. **C)** Comparison of the arm-level CNA between primary and recurrent sites (neutral evolution).

We computed phylogenic trees of the metastatic cascade from the primary to metastatic site (**Fig 2A**). Among 10 CRCR cases (**S1 Table)**, we compared the clonality of mutated driver genes between primary and metastatic sites. We found that most mutated driver genes in primary sites were also observed in metastatic sites, but the mutation allele frequency in several driver genes such as *TP53* (CRCR2), *KRAS* and *PIK3CA* (CRCR3), *TP53* and *CTNB1* (CRCR4) and *KRAS* and *TCFL2* (CRCR5) and *TP53* frameshift (CRCR10) were increased in metastatic sites compared to primary sites (red arrows; **Fig 2B**). The increased cancer cell fraction (CCF) indicated that these mutations in metastatic site might contribute to the progression of metastasis; however, most drivers were mutated as trunk mutations from primary to metastatic sites. Therefore, we considered them as the neutral evolution mutations [9].

Regarding CNAs between nine primary and 10 recurrent sites in 10 CRCR cases, there was no statistically significant difference in CNA frequency between recurrence and primary tumors among all chromosomes (**Fig 2C)**. Therefore, the evolutionary status of CNA from primary to metastatic sites was described as "neutral evolution" because CNAs have contributed to cancer progression as clonal events in both primary and recurrence tumors.

### Difference of the tumor immune response related factors between primary and metastatic sites

**Fig 3:**
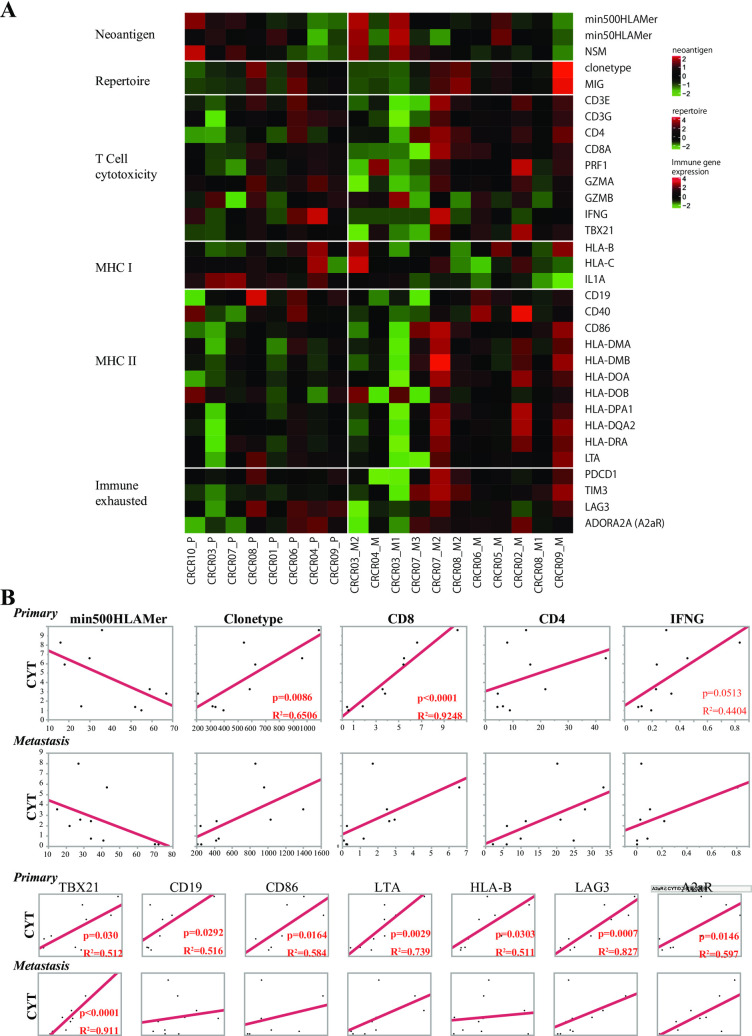
Expression profile of tumor immune responses genes, estimated neoantigens and TCR repertoire diversity. **A)** The top row shows the estimated neoantigen (NAG) level (min500HLAMer, min50HLAMer and non-synonymous mutation), and red indicates the high level of NAG. The second row represents TCR repertoire diversity (#clonotype and #molecular identification group; #MIG), and red indicates heterogeneity intensity. **B)** Association between cytolytic activity (CYT) and tumor immune response-related factors, such as min500HLAMer, TCR repertoire clonotype, CD8, CD4 and IFNG in primary (upper row) and in metastasis (lower row). In addition, CYT activity and expression of *TBX21*, *CD19*, *CD86*, *LTA*, *HLA-B*, *LAG3* and *A2AR* were compared.

To elucidate the association between tumor immune response-related factors in primary and recurrent tumors of stage I and II CRC cases, we examined the expression of the following genes in eight primary sites and 11 recurrence sites. The expression of T cell cytotoxicity (*CD3G*, *CD3E*, *CD4*, *GZMA*, *PRF1* and *TBX21*), B cell MHC II and immune exhausted-related genes were abundant in metastatic sites; while MHC I inflammation related genes (*HLA-B*, *HLA-C* and *IL-1A*) were mostly higher in primary than recurrent tumor sites (**Fig 3A**). Hemon et al. reported that the MHC II was substantially expressed on melanoma-infiltrating T cells with exhaustion [14] concomitantly with immune exhaustion of tumor-infiltrating lymphocytes (TIL) in metastatic lymphocytes.

In addition, we considered not only the expression of each factor or gene but also the cytolytic activity that may be rendered to the actual tumor immune response in confronting cancer progression. We found that the highest difference in correlation coefficient between primary and metastatic tumors was the association between CYT level and the *CD8A* expression **(Fig 3B)**. It is worth noting that the diversity (clonetype) of the TCR repertoire was significantly correlated with the CYT in primary sites. CYT levels were significantly associated with tumor immune response factors, such as *CD19*, *CD86*, *LTA*, *HLA-B*, *LAG3* and *A2AR*, in primary tumors but not in metastatic sites **(Fig 3B, lower row)**. Therefore, we assumed that the cytolytic activity of CTL was disrupted remarkably in metastatic sites in comparison to primary sites.

### Essential factors to determine the recurrence in metastatic sites

As the most important factor in cancer microenvironment might determine the prognosis of cases, we focused on essential factors associated with the relapse-free survival (RFS) period. We had four CRC cases with multiple recurrent sites; however, the RFS period in each recurrent tumor was calculated as the time between the date of operation of the primary sites and each independent recurrent site (**Fig 2A**). SNVs in M1 and M2 in CRCR3 were independent of each other according to the genomic profile; therefore, M2 did not originate from M1 but from the primary site. In CRCR7, M2 and M3 were not from M1 but from the primary site; M2 was not from M1 but from the primary site in CRCR8; and M2 was from the primary site in CRCR10. Notably, 11 recurrent cases relapsed postoperatively in 1.1–35.6 months (average 13.59 ± 9.917 months). The relapse-free survival period was significantly associated with the expression of tumor immune response-related genes, such as *IL1A*, molecular identification group (MIG), TCR repertoire clonetype, and *HLA-C* expression, based on both Pearson_p-value and logrank_p-value **(Table 1)**. Therefore, we focused on the prognostic significance of the TCR repertoire clonetype and the antigenicity of cancer cells in the front line of cancer microenvironment in metastatic sites.

**Table 1:** Correlation between tumor immune related factors in recurrent sites and the relapse free survival period (days). The correlation coefficient, Pearson_p_value and logrank_p_value are described; the statistically significant value is highlighted in yellow.

factor	correlation	pearson_p value	logrank_p_value
**IL1A**	0.7538	0.0118	0.0471
NAG (min500HLAMer)	0.7032	0.0233	ns.
MIG*	-0.6797	0.0306	0.0127
NSM	0.6682	0.0347	ns.
**TCR Clonetype**	-0.6608	0.0375	0.0127
**HLA-C**	0.6514	0.0413	0.0471
IFNG	-0.1365	ns.	0.0064
LTA	-0.5012	ns.	0.0127
CCR4	-0.4529	ns.	0.0127
IL2RA	-0.4375	ns.	0.0197
CXCL13	-0.2278	ns.	0.0197
HLA-DRA	-0.5410	ns.	0.0269
FOXP3	-0.4817	ns.	0.0269
CD3E	-0.4611	ns.	0.0269
GZMA	-0.4503	ns.	0.0269
CYT	-0.4470	ns.	0.0269
HLA-DOA	-0.4369	ns.	0.0269
CD3G	-0.4200	ns.	0.0269
CD8A	-0.3795	ns.	0.0269
CD3D	-0.2933	ns.	0.0269

NAG: neoantigen, MIG; Molecular identified group, ns.; not significant.

### Impaired cytotoxicity associated T-cell receptor repertoire in recurrent sites

**Fig 4:**
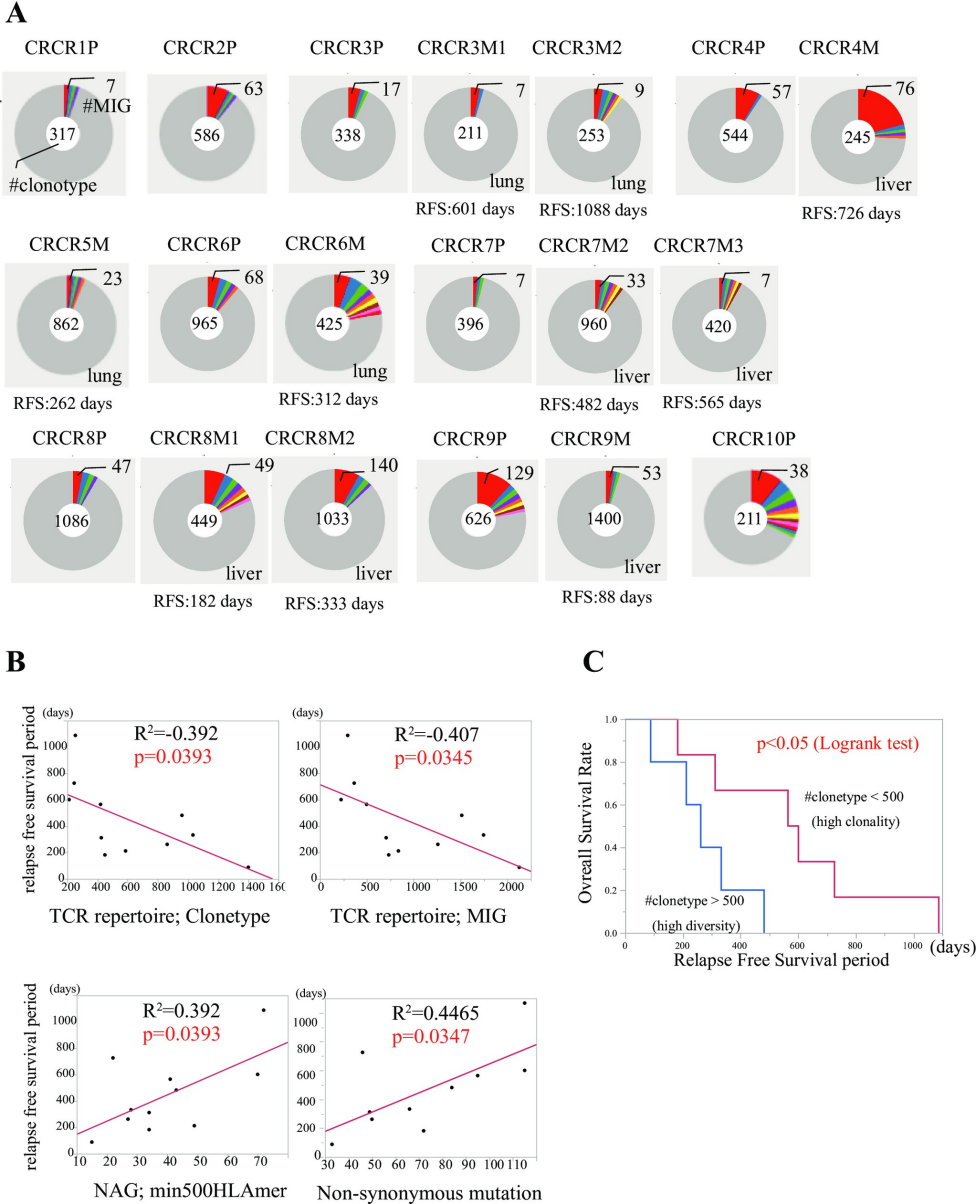
Diversity scores of T-cell receptor (TCR) repertoire in primary and recurrent sites. **A)** TCR repertoires in whole samples were measured with diversity scores in primary and recurrent sites, which are indicated in the center of the circle. #MIG indicates the molecular identification group that represents the number of main repertoire clones. RFS indicates the postoperative recurrence-free period (days). **B)** Association between RFS period and TCR repertoire (clonetype and MIG) (upper row). RFS and neoantigen (min500HLAMer and non-synonymous mutation) (lower row). **C)** comparison of overall survival rate between high (blue) and low clonetype (red).

TCR chain contains hypervariable loops in three complementarities determining regions (CDR). CDR3 is highly variable, and Fang et al. compared sequences between TCR alpha and TCR beta, and showed that the diversity of TCR beta is generated by a somatic recombination process of variable (V), diversity (D) and joining (J) exons, termed the V(D)J recombination. On the other hand, TCR alpha is consisted of VJ recombination [15]. Therefore, we expected that TCR beta could indicate the much higher diversity of TCR than TCR alpha and implemented the evaluation of the TCR repertoire diversity based on TCR beta in the current study. Each CDR3 nucleotide sequence was termed "clonetype". The number of clonetypes (#clonetype) and the number of molecular identified groups (#MIG) in 9 primary tumors and 10 metastatic tumors are presented in **Fig 4A**. Therefore, we verified the association between TCR repertoire clonetype and the expression of CTL related factors in metastatic sites and primary sites (**Table 2**). We observed significant associations mostly in primary sites (78.6%; 11 out of 14 factors) in comparison to the metastatic sites (7.1%; 1 out of 14 factors). The clonetype of TCR repertoire and MIG had a significant inverse association with RFS period, while the antigenicity (NAG; min500HLAmer) and frequency of non-synonymous mutations were significantly associated with RFS (**Fig 4B**). In recurrent tumors, five tumors with higher (diverse) clonetype of TCR repertoire showed poorer prognosis than the five tumors with lower (clonal) clonetype of TCR repertoire (p<0.05) (**Fig 4C**). Comparison of the clonetype between primary and metastatic tumors in two representative cases is shown in **S2 Fig**. CRCR4 showed a long postoperative RFS (24.2 mo) and low diversity in recurrence, and an identical specific clone (CATSMRTGDYNEQFF, TRBV15/TRBJ1-7) was observed. In contrast, CRCR9 relapsed immediately (RFS; 2.9 mo), and diverse clonetypes were observed in the recurrent site.

**Table 2:** Association between TCR repertoire clonetype and CTL related factors in metastatic sites (left) and in primary sites (right). Significant correlation is written in red.

	Clonetype; Metastatic sites (11)		Clonetype; Primary sites (8)	
	R^2^	p-value	R^2^	p-value
NAG (min500mHLAmer)	0.3528	0.0540	0.1983	0.2688
CYT	0.3009	0.0806	0.6709	0.0129
PDL1	0.0054	0.4897	0.4543	0.0668
PD-1	0.3203	0.0795	0.7320	0.0067
CTLA4	0.3505	0.0550	0.9077	0.0003
FOXP3	0.6614	0.0023	0.8584	0.0009
CCR4	0.1776	0.1967	0.6692	0.0131
IL2RA	0.1954	0.1734	0.8909	0.0004
CD4	0.2680	0.1028	0.5464	0.0361
CD8A	0.3788	0.0438	0.7978	0.0028
CD8B	0.0803	0.3756	0.1105	0.4139
CD3D	0.3660	0.0511	0.8508	0.0011
CD3E	0.4885	0.0167	0.9057	0.0003
CD3G	0.3100	0.0752	0.5126	0.0458

### CMS classification and the susceptibility to metastasis

We validated the feasibility of our data by grouping 19 samples into the consensus molecular subtype (CMS) classification [16], which is broadly authorized as a current and comprehensive indicator of the immune infiltration related gene expression and genomic profiles, such as SNV and can, in CRC tissues. In four groups, CMS1 represents the MSI immune type carrying hypermutation, BRAF mutation, and immune infiltration and activation. Three samples, CRCR7M2, 8M1 and 8M2 from metastatic sites were classified into CMS1, while the corresponding primary sites of all three metastatic sites belonged to CMS3, which consisted of tumors with mixed MSI status, low copy number, and KRAS mutation **(Table 3)**. We had 11 recurrent sites, and three metastatic tumors (CRCR7 M2 and 8M1/ M2) out of these were treated with chemotherapies, mFOLFOX6 and UFT/LV. These combination therapies might have induced the infiltration of T-cells into the cancer microenvironment, which might cause the classification of the three cases as CMS1 type by the CMSclassifier (Random forest) [16]. The accumulation of T-cells in cancer microenvironment were derived not by genomic aberrations in CRCR7M, 8M1 and 8M2, but by the exposed combined chemotherapy. Therefore, we conclude that CMS1 phenotype did not cause the recurrence, but the chemotherapy treated 3 recurrent tumors were just classified into the CMS1 trait. As a matter of fact, immune exhaustion markers were abundantly expressed in metastatic sites; however, they were not significantly correlated with the RFS period postoperatively. Therefore, the most critical factors for establishing the recurrence of all tumor immune response elements were the TCR repertoire diversity in CTLs **(Fig 4B and 4C and Table 1),** rather than the accumulation of infiltrating T-cells, such as 3 CMS1 tumors which includes accumulated exhausted T-cells concordantly. However, the number of samples were limited and furthermore studies are required to validate these hypotheses.

**Table 3:** Probability of consensus molecular subtypes (CMS) in 19 current colorectal cancer cases.

	CMS1.posteriorProb	CMS2.posteriorProb	CMS3.posteriorProb	CMS4.posteriorProb	nearest CMS
CRCR07M2	0.41	0.04	0.16	0.39	CMS1
CRCR08M1	0.42	0.21	0.11	0.26	CMS1
CRCR08M2	0.45	0.11	0.14	0.3	CMS1
CRCR03M1	0.05	0.75	0.16	0.04	CMS2
CRCR03M2	0.1	0.65	0.13	0.12	CMS2
CRCR03P	0.05	0.48	0.46	0.01	CMS2
CRCR06M	0.08	0.73	0.04	0.15	CMS2
CRCR09P	0.08	0.68	0.21	0.03	CMS2
CRCR01P	0.17	0.38	0.38	0.07	CMS2,CMS3
CRCR04M	0.07	0.45	0.03	0.45	CMS2,CMS4
CRCR04P	0.22	0.33	0.34	0.11	CMS3
CRCR07P	0.13	0.08	0.79	0	CMS3
CRCR08P	0.06	0.44	0.45	0.05	CMS3
CRCR10P	0.3	0.04	0.66	0	CMS3
CRCR02P	0.37	0.01	0.02	0.6	CMS4
CRCR05M	0.12	0.22	0.04	0.62	CMS4
CRCR06P	0.17	0.23	0.07	0.53	CMS4
CRCR07M3	0.36	0.02	0.04	0.58	CMS4
CRCR09M	0.14	0.38	0.08	0.4	CMS4

### Abundant expression of immune exhaustion related genes in metastatic sites

**Fig 5:**
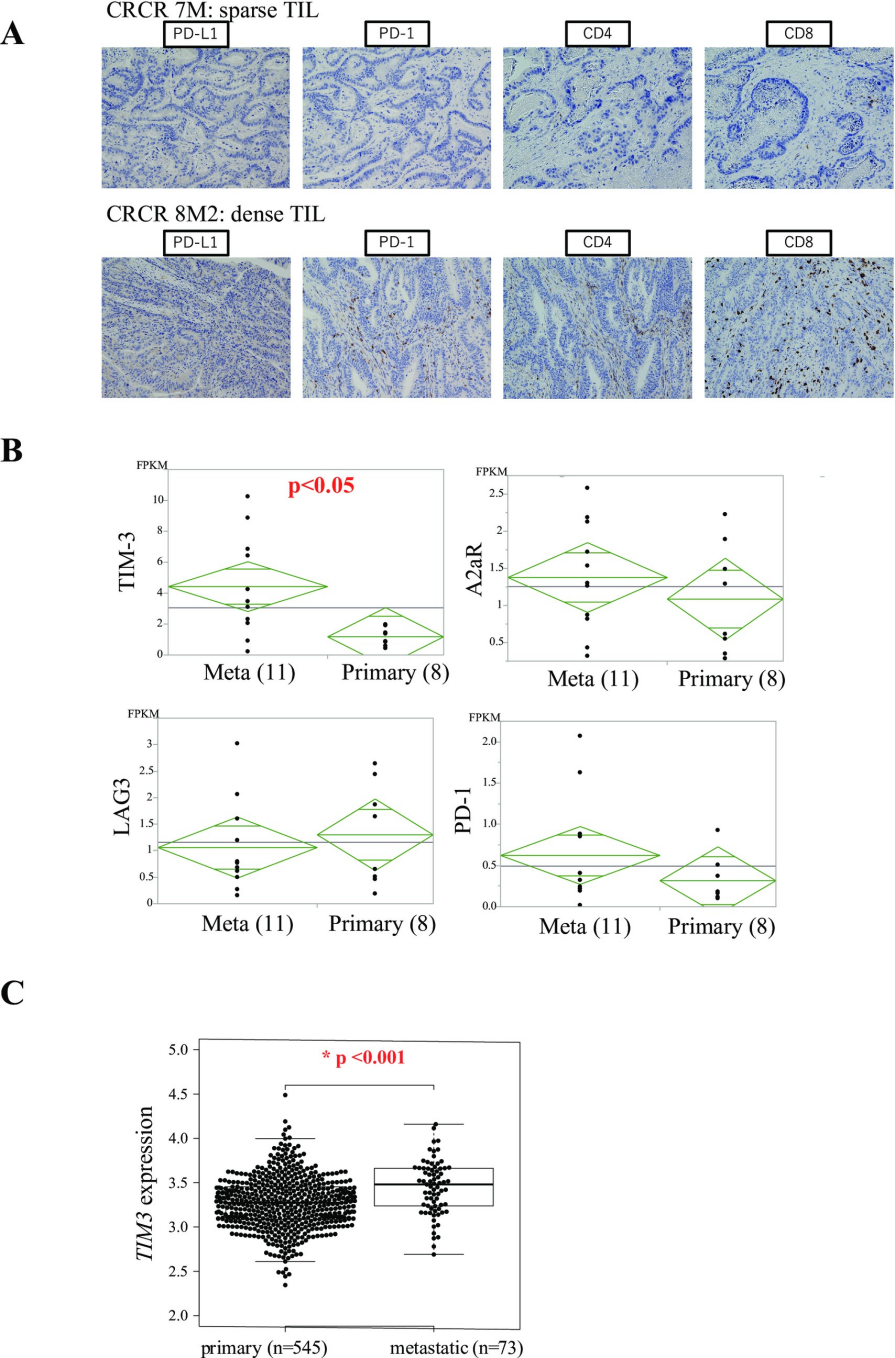
Immunohistochemistry demonstrates the exhaustion of tissue infiltrating lymphocytes. **A)** Protein expression observed in the cytoplasm of infiltrating lymph nodes surrounding cancer cells in primary tumors. The upper row shows a representative image of negative expression (CRCR7), and the lower row shows positive expression with infiltrating T cell accumulation in primary tumors (CRCR8). **B)** The difference in expression of T cell exhaustion molecules, such as *PD-1*, *LAG3*, *TIM3* and *A2aR* between primary sites and metastatic sites. **C)** *TIM3* expression in primary sites and metastatic sites (TCGA data).

CRCR7 and CRCR8 were representative cases indicative of the lower and higher expression of T cell exhaustion markers PD1, respectively, in CD8 or CD4 **(Fig 5A)**. We validated the significant association between the expression of CD8 and PD-1 in both primary and metastatic tissues by immunohistochemical study. PD-1 expression indicated an immune exhaustion in CD8 expressing T cells. We found the most significant association between CD8 and PD-1 (p<0.0001) (**S3 Fig**). Along with the increased number of infiltrating T-cells, expression of immune exhaustion markers, such as PD-1 and TIM3 were accumulated concordantly. We examined expression of T cell exhaustion-related genes, such as *TIM-3*, *A2AR*, *LAG3* and *PD-1* in metastatic and primary tumors **(Fig 5B)**. It is worth noting that *TIM3* expression was more abundant in metastatic sites than in primary tumors by RNA sequencing analysis (t-test, p < 0.05) **(Fig 5B)**, which was consistent with the TCGA dataset (p<0.001) (**Fig 5C**). Based on the IHC results, the abundant expression of immune exhaustion markers, such as PD-1 and TIM3, were concordant with the increased number of exhausted CTLs. According to Table 1, however, those immune exhaustion markers were not prognostic markers of postoperative recurrence.

## Discussion

The current study focused on the selective pressures for cancer metastasis from stage I or II CRC primary tumors to metastatic tumors. We previously reported that CNAs might play crucial roles in the evolution of primary cancer [9], and we hypothesized that genomic aberration might play an important role in the onset of metastasis. In this study of the comparison of primary tumors between CRCR and PCRC, we found a significant inverse association between CNAs, particularly in clonal amplified arms (20q, 7p, 13q and 7p), similar to the tumor immune response reported in CRC cases by Taylor et al [13]. Reduced NAGs with abundant CNAs in primary sites were significantly related to a higher incidence of tumor recurrence, even after curative operation. We speculated that CNAs at the arm level would give rise to the elimination of clones with abundant antigenicity through activated CTL attack. Therefore, amplified arms in primary sites may activate oncogenes on those chromosomes and acquire immune evasion by reducing antigenicity, consistent with previous reports [12,17].

Based on these results, we believed at first that metastatic sites might accumulate multiple CNAs or driver mutations in comparison to primary sites. However, the CNA level did not segregate the metastatic capability in primary or recurrent sites, nor did they present recurrence-specific genomic alterations. In the current study, we focused on the tumor immune-related factors that significantly affected RFS period. We found that TCR repertoire diversity (clonetype and MIG) in recurrent tumors was one of the most important factors. Therefore, the microenvironment of the antigen-antibody interaction that confronts cancer progression in recurrent sites might be one of the most crucial elements that decide susceptibility to the metastasis. In comparison of the association between TCR repertoire and CTL-related molecules in primary and in recurrent tumors, we found a robust association in primary site than in recurrent sites, suggesting that the diminished tumor immune response was an inevitable event for establishing cancer metastasis. In addition, the increased number of clones with T-cell exhaustion molecules, such as *PD-1* and *TIM3*, impaired the host immunity against tumor progression. However, we found that *PD-1* and *TIM3* were not prognostic factors, while the maximized NAG and the diverse TCR repertoire clonetype were significantly associated with RFS period. Therefore, study focus should be on the front line of the battle field in the cancer microenvironment between NAG in cancer cells versus TCR repertoire of CTLs to control the cancer metastasis, rather than on other tumor immune response molecules, such as T-cell exhaustion molecules *PD-1* and *TIM3*.

**Fig 6:**
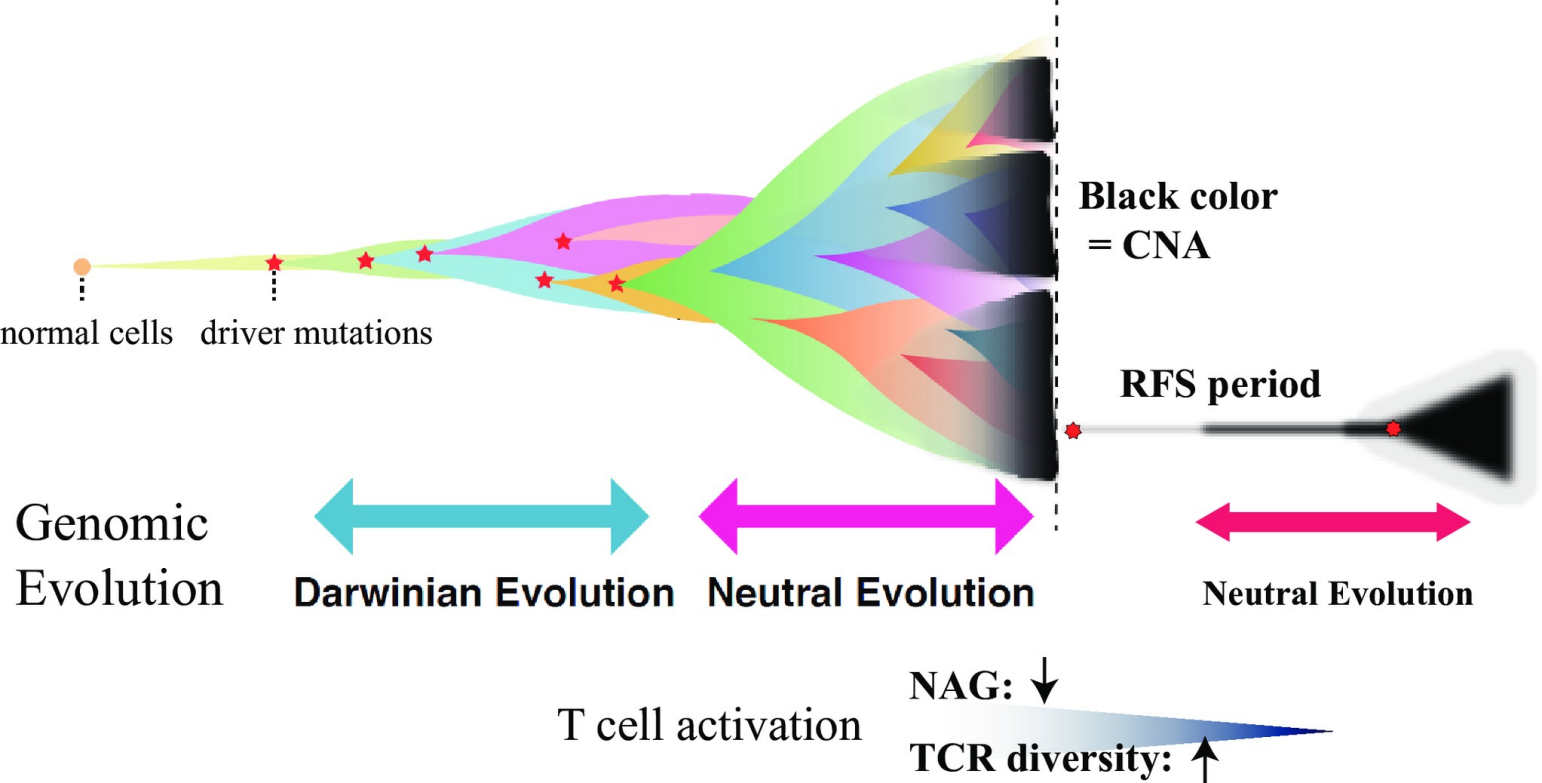
A proposed model for the evolution from primary to metastasis in early CRC cases. This figure is a modified version of our previously reported model [9]. In primary tumors with postoperative recurrence, there is an abundance of CNA and the altered aberrant chromosome might induce a diminished tumor immune response (i.e. reduced T cell activation). Both persistent CNA and the trunk driver mutations indicate neutral evolution of primary to recurrence in tumors. The tumor immune response is observed as reduced neoantigens and clonality of TCR repertoire as selective pressures that might affect neutral evolution events in recurrent tumor sites.

In terms of cancer evolution, we conclude that genomic progression from primary to recurrent tumor shapes the “neutral evolution” of these tumors, since CNA from primary to recurrence was not altered and exacerbated during the metastatic process. Nevertheless, the increased mutation allele frequency of driver genes in metastatic sites were mostly trunk mutations from primary to metastasis (**Fig 2A**), which is consistent with “neutral evolution” (**Fig 6**). During the neutral evolution of cancer genome, tumor immune response related factors (NAG and TCR repertoires) in metastatic sites rather than primary tumor sites were the selective pressures for metastasis in colorectal cancers.

There were some limitations in this study. For example, it was difficult to determine the significance of the TCR repertoire without accurately and comprehensively sequencing the CDR regions of all four types (α, β, γ, and δ) of TCR chains [18]. Furthermore, we did not conduct RNA sequencing of PCRC samples because it was difficult to simultaneously obtain adequate amounts of total RNA and genomic DNA from small amounts of materials. Moreover, it was necessary to examine additional cases to conclude that TCR repertoire and NAG levels were more critical markers than genomic aberrations and CNAs in determining metastasis. However, there are no adequate data or interpretation for the dissociation of diminished tumor immune responses and genomic aberrations, such as aneuploidy of chromosomes 7, 8, 13, and 20 and LOH in chromosome 6, which encodes MHC class I [19]. Further study is required to overcome these limitations.

In conclusion, we found that CNAs may function as the primary selective pressure that promotes cancer evolution from early to advanced tumors in primary sites. Diminished NAG in cancer cells and the diverse TCR repertoire in cytotoxic T cells were crucial condition for provoking postoperative recurrence during the second genomic neutral evolution from primary sites to recurrent sites. Therefore, we could prevent cancer metastasis by activating CTL at premetastatic sites before the priming of the metastatic process.

## Materials and methods

### Ethics statement

The study design was approved by the institutional review boards and ethics committees of the hospitals to which the patients were admitted (Kyushu University Hospital Institutional Review Board: protocol number 2010–1058; Cancer Institute Hospital Institutional Review Board: protocol number 12–27). The study was conducted according to the principles expressed in the Declaration of Helsinki. We obtained written informed consent from all participants in this study.

### Enrolled patients

We performed multiregional WES on 10 early (stage I or II) colorectal tumor cases with postoperative recurrence (**S1 Table**). In addition, we applied CRC data from The Cancer Genome Atlas (TCGA) database as follows [20].

### Sample collection and preparation

Genomic DNA and RNA were extracted from freshly frozen tumor samples and adjacent normal intestinal mucosa using an All Prep DNA/RNA Mini Kit (Qiagen, Hilden, Germany) according to the manufacturer’s instructions.

### TCR repertoire analysis

We synthesized and amplified TCR beta sequences with sample barcodes from RNA extracted from freshly frozen tumor samples as previously described [19]. Captured DNA was sequenced using MiSeq (Illumina, San Diego, CA, USA) with the paired-end 150-bp read option. Sequencing data were analyzed using the MIGEC tool [21] and VDJtools [22].

### Whole exome sequencing

DNA was captured using a SureSelect Human All Exon 50 Mb kit (Agilent Technologies, Santa Clara, CA, USA) according to the manufacturer’s instructions. Captured DNA was sequenced using a HiSeq 2500 (Illumina) with the paired-end 75–100-bp read option. Information on read depth is provided in **S4 Table**.

### Copy number aberrations

DNA was processed and hybridized to the Human Omni Express Bead Chip Kit (Illumina). Illumina’s GenomeStudio software was used to obtain B allele frequencies (BAFs) and log R ratios (LRRs) from the raw output data. BAFs and LRRs were put into the ASCAT algorithm [23] to estimate purity and allele-specific absolute CN. If DNA quality was too poor to use the above kit, we detected CNAs from WES data using the software tool EXCAVATOR (http://sourceforge.net/projects/excavatortool/)) [24], and the data were then applied to subsequent analyses.

### Mutation calling

Sequence data were processed through an in-house pipeline (https://genomon-project.github.io/GenomonPagesR/). The sequencing reads were aligned to the NCBI Human Reference Genome Build 37 hg19 with BWA version 0.7.8 using default parameters. Polymerase chain reaction duplicate reads were removed with Picard. Mutation calling was performed using the EBcall algorithm [25] with the following parameters: 1) mapping quality score ≥ 20, 2) base quality score ≥ 15, 3) both the tumor and normal depths ≥ 10, 4) variant reads in tumors ≥ 4, 5) variant allele frequencies (VAFs) in tumor samples ≥ 0.02, and 6) VAFs in normal samples ≤ 0.01.

### Prediction of neoantigen (NAG), HLA genotyping (Hayashi Method)

For HLA genotyping from whole-genome sequencing data, we used the Bayesian ALPHLARD method, which is designed to perform accurate HLA genotyping from short read data together with predicting HLA sequences of the sample. The latter function enabled identification of somatic mutations by comparing HLA sequences of the tumor and matched-normal samples. The statistical formulation for the posterior probability is described as *P*(*R,S,I|X*)∝*P*(*X|S,I*)*P*(*I*)*P*(*R,S*), where *R* = (*R*_1_,*R*_2_) is the pair of HLA types (reference sequences), *S* = (*S*_1_,*S*_2_) is the pair of sample HLA sequences, *X* = (*x*_1_,*x*_2_,…) is the set of sequence reads, and *I* = (*I*_1_,*I*_2_,…) is the set of variables taking one or two (the j^th^ element, I_j_, indicates that the j^th^ read x_j_ is generated from $S_{I_{j}}$). On the right-hand side of the equation, the left term indicates the likelihood of the sequence reads when HLA and reference sequences are fixed. The middle and right terms are the priors. The parameters, HLA sequences, and HLA types were determined by the MCMC procedure.

### Prediction of potential NAGs by Neoantimon

Using the above statistical method, we obtained the HLA types of individual patients. Then, to identify potential NAGs, we used a non-relapsed-based automated pipeline available at https://github.com/hase62/Neoantimon. From WES data, this pipeline can easily and automatically construct mutated and wild-type peptides, including mutation position, calculate their binding affinities to major histocompatibility complex (MHC) molecules (by netMHCpan3·0), and integrate the total and tumor-specific RNA expression data based on VAFs at the mutation position.

### RNA Sequencing (RNA-Seq)

We sequenced cDNA fragments from a population of reverse-transcribed RNA extracted from eight primary sites and 11 metastatic sites in 10 CRCR cases. Primary in CRCR2 and CRCR5, metastatic sites in CRCR1, CRCR7 and CRCR10 yielded an inadequate amount of RNA and were excluded from analysis. We generated up to three billion single-end reads on an Illumina HiSeq 2500 system as described previously [26].

### Evaluation of cytolytic activity (CYT) and tumor-infiltrating lymphocyte-related molecules

To evaluate the tumor immune response level in clinical tissue samples, we examined CYT [26,27], calculated as the geometric mean of *GZMA* and *PRF1* expression values, since both molecules are tightly co-expressed in TCGA samples [19]. *GZMA* and *PRF1* are dramatically upregulated upon CD8+ T cell activation [28].

### Immunohistochemistry (IHC)

IHC was performed in 19 formalin-fixed paraffin-embedded (FFPE) sections prepared from 8 primary and 11 metastatic lesions. After antigen retrieval, sections were incubated with primary antibodies: anti-programmed cell death ligand 1 (PDL1; ab205921; Abcam, Cambridge, UK), anti-programmed cell death 1 (PD1; ab52587; Abcam), anti-CD4 (713951; Nichirei Biosciences Inc., Tokyo, Japan), and anti-CD8 (413201; Nichirei Biosciences Inc.). Antigen-antibody complexes were visualized using horseradish peroxidase-conjugated secondary antibodies and diaminobenzene. Sections were randomized and analyzed by two blinded, independent observers.

### TCGA dataset

We downloaded mRNA expression data and clinical assessments of 623 patients with CRC from the Broad Institute’s Firehouse (gdac.broadinstitute.org_COADREAD.mRNAseq_Preprocess.Level_3.2016012800.0.0; gdac.broadinstitute.org_COADREAD.Merge_Clinical.Level_1.2016012800.0.0). We normalized mRNA expression data (fragments per kilobase of transcript per million mapped reads (FPKM), raw count) using the quantile normalization method, which is a technique for making two distributions identical in statistical properties. We also log10-transformed normalized data before subsequent analyses.

### Consensus molecular subtype (CMS) classification of Nagayama samples

Firstly, RNA-seq of 19 CRC samples (primary and metastatic tissues), called Nagayama samples, were performed using Illumina HiSeq 2500 platform. The fastq files of RNA-seq reads were aligned to the human reference genome, and genes were annotated (UCSC hg19) using TopHat v2.0.12. Cufflinks v2.2.1 was used to calculate the FPKM values of each gene. Secondly, CMS classification of Nagayama samples was performed using CMSclassifier (Random forest), which is an R package reported previously [16]. The input file of the CMSclassifier was a matrix file consisting of the FPKM values of each gene from Nagayama samples. CMS of each CRC sample was predicted by the CMSclassifier.

### Statistical analysis

Associations between variables were tested with Mann-Whitney U-tests or Fisher’s exact tests. The degree of linearity was estimated by Pearson’s correlation coefficient. Overall survival (OS) was estimated using the Kaplan-Meier method, and survival curves were compared using a log-rank test. A two-sided *p* < 0.05 was considered significant. Data analyses were performed using JMP 14 software (SAS Institute, Cary, NC, USA) and R software version 3·1·1 (The R Foundation).

## Supporting information

S1 Fig Clinical significance of cytolytic activity in CRC cases. **A)** Comparison of overall survival (left) and recurrence free survival (right) between CYT high and CYT low in stage I or II CRC cases of TCGA. **B)** Comparison of overall survival between CYT high and CYT low of all CRC cases in TCGA.
(PDF)

S2 Fig Comparison of CDR3 amino acid sequence between primary and recurrent tumors in two representative cases. **A)** CRCR4; relapse-free survival (RFS) period (24.2 mo). Amino acid CATSMRTGDYNEQFF, TRBV15/TRBJ1-7 clone is identical between primary and recurrent tumors. **B)** CRCR9; RFS period (2.9 mo). Diversity is expanded and no identical clone was observed between primary and recurrent tumors.
(PDF)

S3 Fig Immunohistochemical study by monoclonal antibodies CD8, CD4 PD-1 and PD-L1 for 8 primary and 11 metastatic tissues. We validated that the immune exhaustion has been observed in CD8 expressing T cell in CRC tissues.
(PDF)

S1 Table Clinicopathologic information of 10 cases of colorectal cancer with recurrence (CRCR) and 8 cases of precancerous tumors (PCRC). In terms of PCRC, we conducted genomic analysis for all samples, 10 primaries and 15 metastasis, however, there were inadequate amount of RNA, so we could not obtain RNA Sequence data in 2 primary (CRCR2P and CRCR5P) and 4 metastatic tumors (CRCR1M, CRCR7M1, CRCR10M1 and M2).
(PDF)

S2 Table The inverse association between copy number aberrations and single nucleotide variance in the entire chromosomes (TCGA). Significant inverse association (q-value<0.1) was a half area highlighted in blue.
(PDF)

S3 Table Comparative data of the expression levels of tumor immune response-related factors in primary tumors with recurrence (40 cases) and cases without recurrence (232 cases) in TCGA database. Statistically significant value is written in red.
(PDF)

S4 Table Coverage data of the Sequence analysis of whole CRCR cases in Fig 1. (PDF)
