# Supplementary figures and images for: Impaired tumor immune response in metastatic tumors is a selective pressure for neutral evolution in CRC cases
Source: PLoS Genet. 2021 Jan 21;17(1):e1009113. doi: 10.1371/journal.pgen.1009113 (PMC7864431; doi:10.1371/journal.pgen.1009113)

A

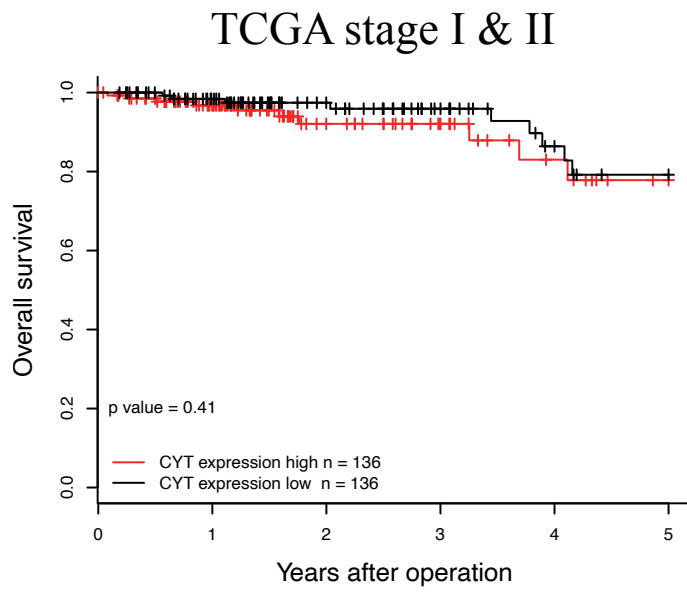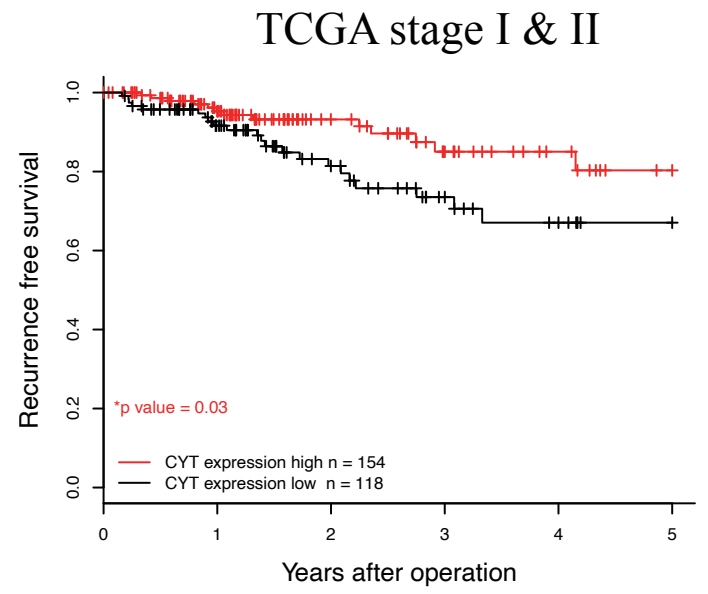

B

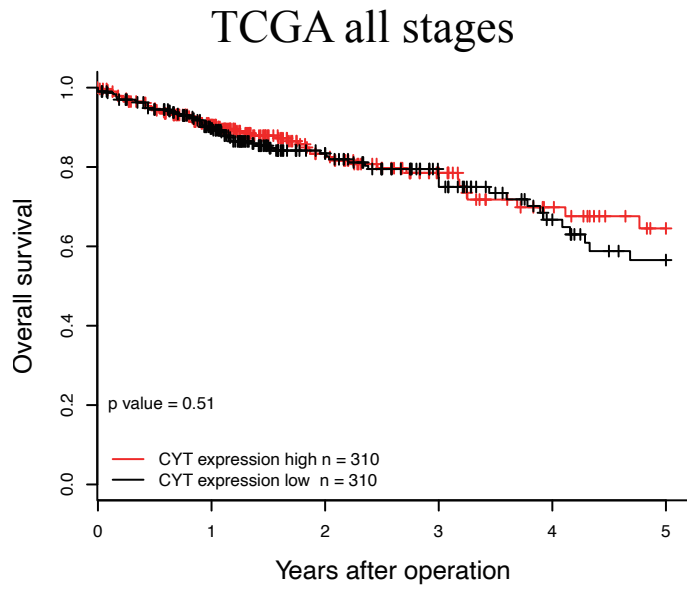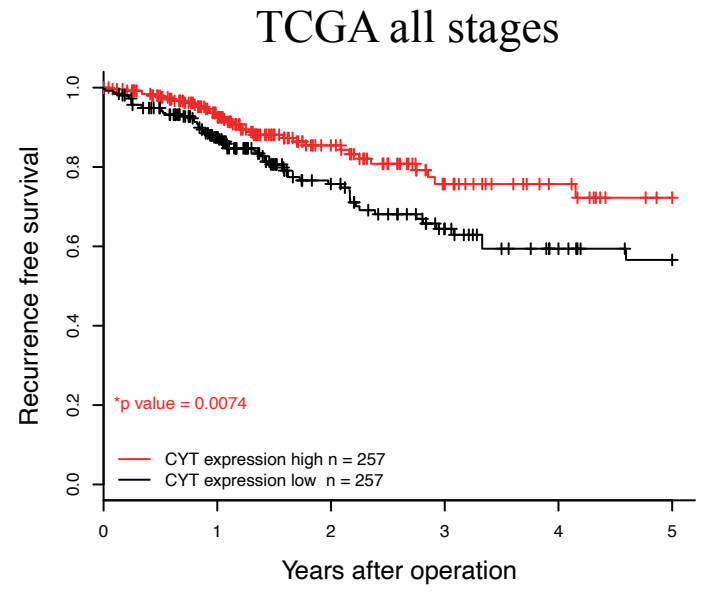

Supplement: S1 Fig — A) Comparison of overall survival (left) and recurrence free survival (right) between CYT high and CYT low in stage I or II CRC cases of TCGA. B) Comparison of overall survival between CYT high and CYT low of all CRC cases in TCGA. (PDF) [file pgen.1009113.s001.pdf]

A

RFS : 24.2M

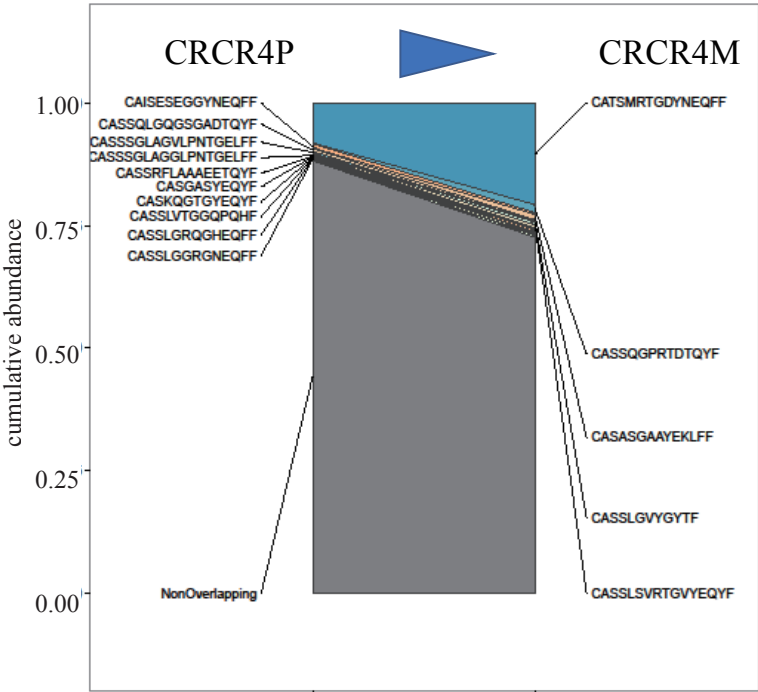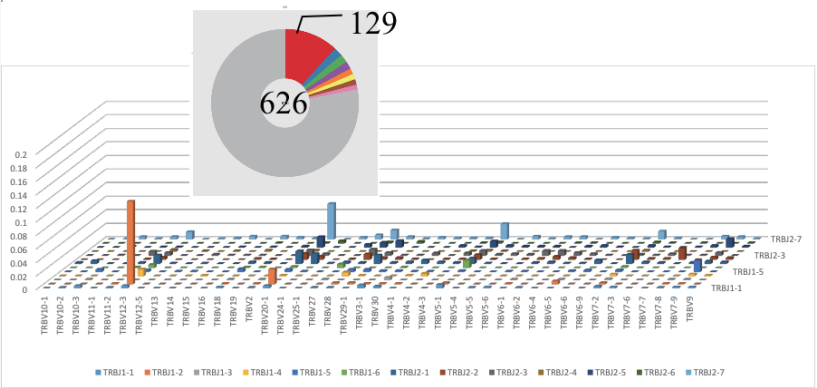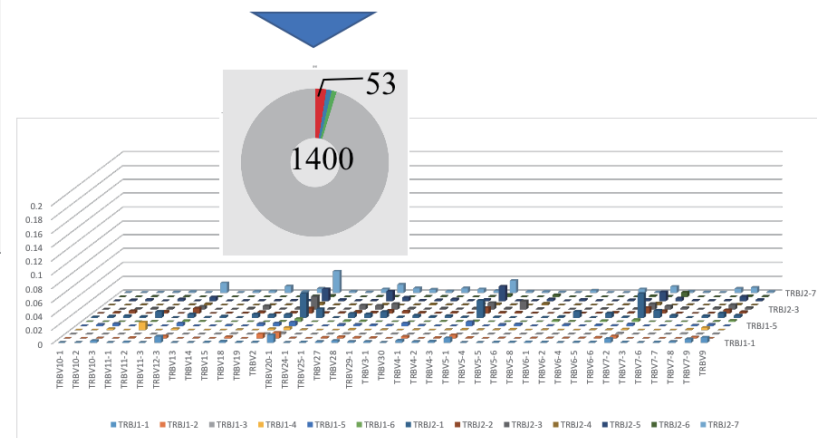

B

RFS: 2.9M

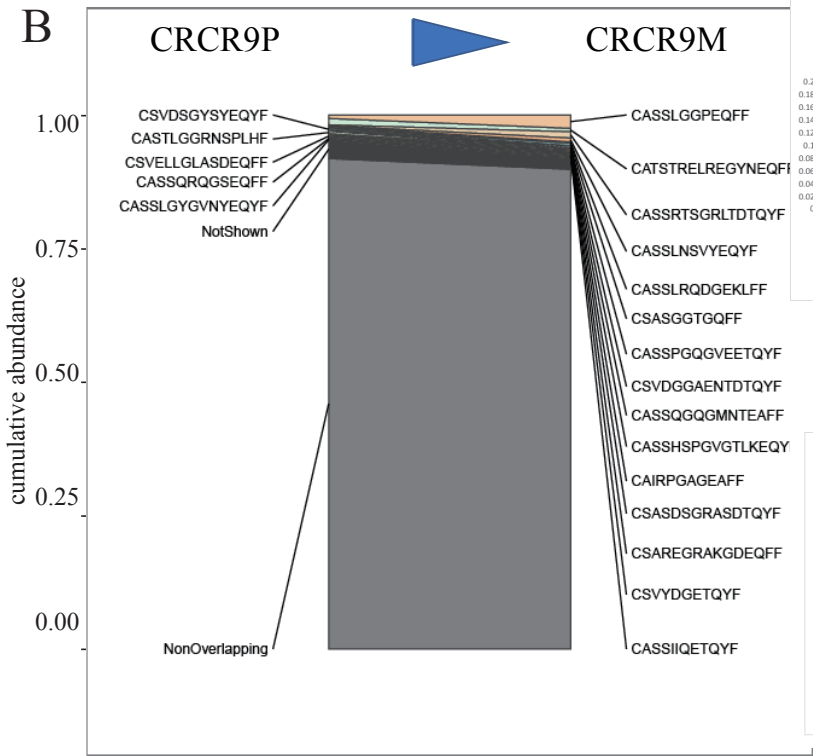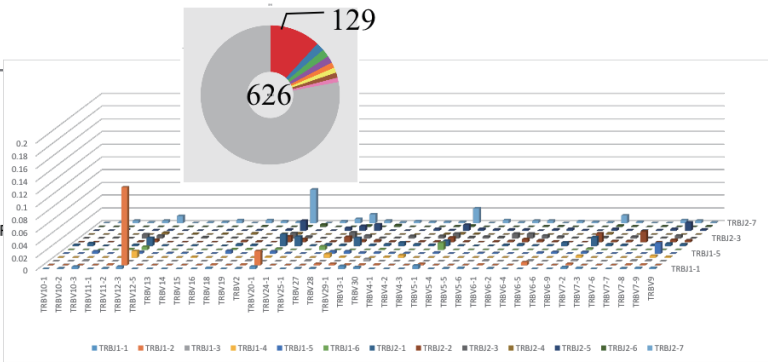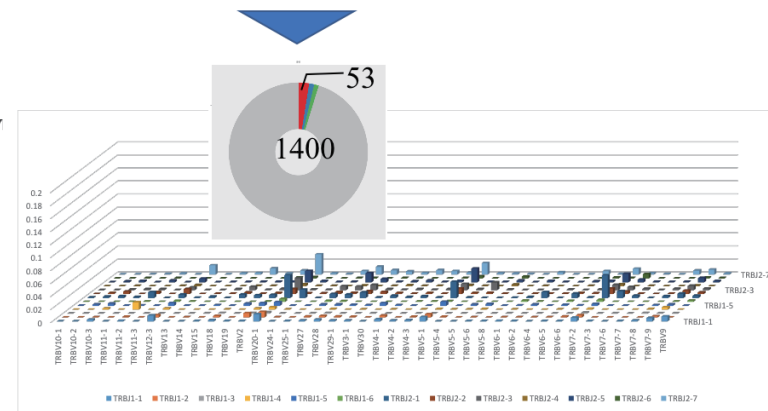

Supplement: S2 Fig — A) CRCR4; relapse-free survival (RFS) period (24.2 mo). Amino acid CATSMRTGDYNEQFF, TRBV15/TRBJ1-7 clone is identical between primary and recurrent tumors. B) CRCR9; RFS period (2.9 mo). Diversity is expanded and no identical clone was observed between primary and recurrent tumors. (PDF) [file pgen.1009113.s002.pdf]

Figure S3

Association between CD8 (T cell) and PD-1 (immune exhaustion marker) expressions.

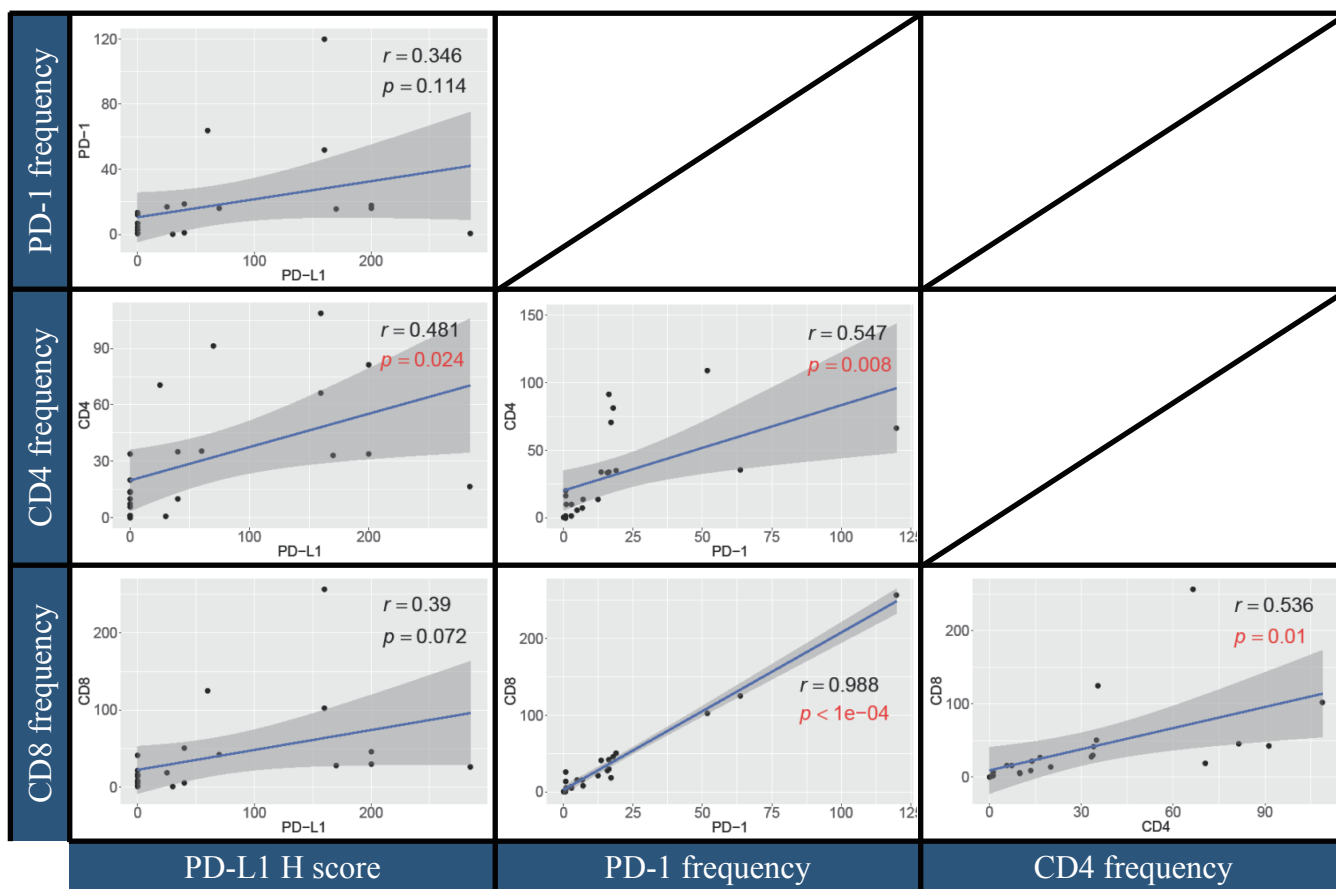

Supplement: S3 Fig — We validated that the immune exhaustion has been observed in CD8 expressing T cell in CRC tissues. (PDF) [file pgen.1009113.s003.pdf]
