# Supplementary material for: Impaired tumor immune response in metastatic tumors is a selective pressure for neutral evolution in CRC cases
Source: PLoS Genet. 2021 Jan 21;17(1):e1009113. doi: 10.1371/journal.pgen.1009113 (PMC7864431; doi:10.1371/journal.pgen.1009113)
Supplement: S1 Table — In terms of PCRC, we conducted genomic analysis for all samples, 10 primaries and 15 metastasis, however, there were inadequate amount of RNA, so we could not obtain RNA Sequence data in 2 primary (CRCR2P and CRCR5P) and 4 metastatic tumors (CRCR1M, CRCR7M1, CRCR10M1 and M2). (PDF) [file pgen.1009113.s004.pdf]

Supplementary Table 1. Information of 10 cases of colorectal cancer with recurrence (CRCR) and 8 cases of precancerous tumors (PCRC).

ID	Sex	Age	primary tumor						metastattic tumor	
			**Tumor location**	**Size**	**Tumor differentiation**	**UICC stage**	**ly**	**v**	**Recrrence site**	**Months after surgery**
CRCR1	M	68	sigmoid colon	40x40	well differentiated	Stage Ⅱ (T4N0M0)	1	1	Liver*	4.1
CRCR2*	F	63	rectum (Rb)	15	well to moderately differentiated	Stage Ⅰ (T1N0M0)	0	0	Lung	8.7
CRCR3	M	33	rectum (Ra)	40x40	well differentiated	Stage Ⅱ (T3N0M0)	1	1	Lung<br>Lung	M1: 20<br>M2: 35.6
CRCR4	M	41	sigmoid colon	23x19	moderately differentiated	Stage Ⅰ (T2N0M0)	1	2	Liver	24.2
CRCR5*	F	60	rectum (Ra)	30x30	well differentiated	Stage Ⅱ (T3N0M0)	1	1	Lung	12.6
CRCR6	M	56	rectum (Rb)	32x28	moderately differentiated	Stage Ⅰ (T2N0M0)	2	1	Lung	10.4
CRCR7	F	73	ascending colon	25x25	moderately differentiated	Stage Ⅰ (T1N0M0)	1	0	Liver*<br>Liver<br>Liver	M1: 5.3<br>M2: 16.1<br>M3: 18.8
CRCR8	M	76	sigmoid colon	28x20	moderately differentiated	Stage Ⅱ (T4N0M0)	1	2	Liver<br>Liver	M1: 6.1<br>M2: 11.1
CRCR9	F	55	sigmoid colon	35x35	moderately differentiated	Stage Ⅱ (T3N0M0)	0	2	Liver	2.9
CRCR10	F	78	rectum (Ra)	20x20	well differentiated	Stage Ⅰ (T2N0M0)	1	1	Lung*<br>Lung*	M1: 6.6<br>M2: 29.7

Genomic analysis was conducted for all samples, 10 primaries and 15 metastasis. * No RNA Sequence data in 2 primary and 4 metastatic tumors.

**ID**	**Sex**	**Age**	**Tumor location**	**Size**	**Tumor differentiation**	**UICC stage**	**ly**	**v**	**Recrrence site**	**Months after surgery (M)**
PCRC01	66	M	Rectum (RS)	35×25mm	adenocarcinoma with adenoma component	pTXN0M0	0	0	none	47
PCRC03	80	F	Cecum	43×34mm	adenocarcinoma in tubular adenoma	Stage0 pTis(M)N0M0	0	0	none	45
PCRC05	81	F	Transvers colon	35×12mm	well differentiated adenocarcinoma with adenomatous component	Stage0 pTis(M)N0M0	0	0	none	42
PCRC06	81	M	Descending colon	28×25mm	adenocarcinoma in adenoma	Stage0 pTis(M)N0M0	0	0	none	42
PCRC08	77	M	Rectum (Ra)	43×35mm	adenocarcinoma in tubular adenoma	pTis(M)N0M0	0	0	none	40
PCRC12	83	M	Rectum (Rb)	33×30mm	adenocarcinoma in tubular adenoma	Stage0 pTis(M)N0M0	0	0	none	38
PCRC13	87	F	Hepatic flexure	53×40mm	adenocarcinoma in tubular adenoma	Stage0 pTis(M)N0M0	0	0	none	38
PCRC15	82	M	Rrectum (Ra)	70×60mm	adenocarcinoma with adenoma component	Stage0 pTis(M)N0M0	0	0	none	33
