# Supplementary material for: Impaired tumor immune response in metastatic tumors is a selective pressure for neutral evolution in CRC cases
Source: PLoS Genet. 2021 Jan 21;17(1):e1009113. doi: 10.1371/journal.pgen.1009113 (PMC7864431; doi:10.1371/journal.pgen.1009113)
Supplement: S2 Table — Significant inverse association (q-value<0.1) was a half area highlighted in blue. (PDF) [file pgen.1009113.s005.pdf]

Supplementary Table 2 The association between the (inverse) association between copy number aberrations and single nucleotide variance (TCGA).

chromosome	pvalue	qvalue*
7p	2.43E-10	5.35E-09
20q	1.46E-10	5.35E-09
20p	1.27E-09	1.86E-08
13p	9.37E-09	1.03E-07
13q	1.47E-08	1.29E-07
7q	1.77E-08	1.30E-07
8q	4.16E-06	2.61E-05
16p	3.34E-05	0.0001837
2q	0.000158	0.000726
16q	0.000165	0.000726
5p	0.000399	0.001596
6q	0.000678	0.002486
2p	0.00111	0.003756923
19q	0.00145	0.004557143
19p	0.00478	0.014021333
3q	0.00608	0.01672
11p	0.0119	0.0308
11q	0.0162	0.037515789
17q	0.0156	0.037515789
5q	0.0174	0.03828
21q	0.03	0.062857143
14q	0.044	0.088
10p	0.0734	0.140417391
12q	0.0772	0.141533333
9p	0.0872	0.153472
1q	0.0999	0.1628
21p	0.0999	0.1628
9q	0.117	0.183857143
14p	0.161	0.2376
18q	0.162	0.2376
12p	0.212	0.300903226
1p	0.238	0.306777778
6p	0.23	0.306777778
8p	0.247	0.306777778
10q	0.24	0.306777778
22p	0.251	0.306777778
22q	0.378	0.449513514
4p	0.451	0.522210526
15q	0.551	0.621641026
17p	0.593	0.6523
4q	0.678	0.727609756
18p	0.772	0.808761905
3p	0.828	0.847255814
15p	0.866	0.866

*The upper half arms showed the statistical significant association betweeen CNA and SNV (q-value).
