# Supplementary material for: Impaired tumor immune response in metastatic tumors is a selective pressure for neutral evolution in CRC cases
Source: PLoS Genet. 2021 Jan 21;17(1):e1009113. doi: 10.1371/journal.pgen.1009113 (PMC7864431; doi:10.1371/journal.pgen.1009113)
Supplement: S3 Table — Statistically significant value is written in red. (PDF) [file pgen.1009113.s006.pdf]

Supplementary Table 3 Comparison of the expression level of tumor immune response related molecules and factors between primary tumors with recurrence (40 cases) and those without recurrence (232 cases) in TCGA database.

Genes or Factor	fold change (Rec*/Non-Rec)	p-value
CCR4	0.811	0.2045
CD25	0.792	0.1457
CD3D	0.787	0.2351
CD3E	0.820	0.1620
CD3G	0.672	0.0199
CD4	0.870	0.2184
CD8A	0.796	0.2168
CTLA4	0.984	0.9792
CYT	0.777	0.0734
FOXP3	0.921	0.5329
GZMA	0.729	0.0692
PD1	0.747	0.0848
PDL1	0.926	0.3900
PRF1	0.862	0.3236

*Rec.= recurrence
