# Supplementary material for: Impaired tumor immune response in metastatic tumors is a selective pressure for neutral evolution in CRC cases
Source: PLoS Genet. 2021 Jan 21;17(1):e1009113. doi: 10.1371/journal.pgen.1009113 (PMC7864431; doi:10.1371/journal.pgen.1009113)
Supplement: S4 Table — (PDF) [file pgen.1009113.s007.pdf]

**Supplementary Table 4 Coverage of the Sequence analysis of CRCR cases**

| | | | Depth | | | |
|---|---|---|---|---|---|---|
| | | | mean coverage | >10x fraction | >20x fraction | >30x fraction |
| CRCR 1 | normal | frozen | 86.6 | 0.985 | 0.948 | 0.884 |
| | primary | frozen | 156.4 | 0.994 | 0.982 | 0.962 |
| | meta | frozen | 135.5 | 0.988 | 0.962 | 0.921 |
| CRCR 2 | normal | frozen | 64.9 | 0.975 | 0.907 | 0.803 |
| | primary | FFPE | 45.8 | 0.937 | 0.791 | 0.627 |
| | meta | frozen | 90.0 | 0.984 | 0.949 | 0.888 |
| CRCR 3 | normal | frozen | 71.8 | 0.978 | 0.921 | 0.83 |
| | primary | frozen | 97.6 | 0.986 | 0.954 | 0.899 |
| | meta1 | frozen | 181.3 | 0.994 | 0.985 | 0.969 |
| | meta2 | frozen | 64.6 | 0.972 | 0.897 | 0.790 |
| CRCR 4 | normal | frozen | 112.2 | 0.991 | 0.97 | 0.931 |
| | primary | frozen | 92.1 | 0.988 | 0.955 | 0.899 |
| | meta | frozen | 111.5 | 0.988 | 0.959 | 0.913 |
| CRCR 5 | normal | frozen | 92.9 | 0.987 | 0.956 | 0.901 |
| | primary | FFPE | 61.1 | 0.974 | 0.907 | 0.800 |
| | meta | frozen | 84.7 | 0.982 | 0.937 | 0.865 |
| CRCR 6 | normal | frozen | 60.4 | 0.969 | 0.888 | 0.771 |
| | primary | frozen | 102.2 | 0.987 | 0.958 | 0.909 |
| | meta | frozen | 82.8 | 0.982 | 0.933 | 0.856 |
| CRCR 7 | normal | frozen | 164.0 | 0.994 | 0.984 | 0.967 |
| | primary | frozen | 95.2 | 0.987 | 0.957 | 0.905 |
| | meta1 | FFPE | 62.0 | 0.971 | 0.893 | 0.782 |
| | meta2 | frozen | 71.6 | 0.976 | 0.916 | 0.823 |
| | meta3 | frozen | 143.7 | 0.991 | 0.975 | 0.947 |
| CRCR 8 | normal | frozen | 138.8 | 0.994 | 0.980 | 0.955 |
| | primary | frozen | 140.5 | 0.994 | 0.98 | 0.956 |
| | meta1 | frozen | 96.3 | 0.985 | 0.945 | 0.881 |
| | meta2 | frozen | 77.2 | 0.977 | 0.921 | 0.833 |
| CRCR 9 | normal | frozen | 92.0 | 0.985 | 0.952 | 0.895 |
| | prmary | frozen | 104.8 | 0.988 | 0.960 | 0.913 |
| | meta | frozen | 46.3 | 0.943 | 0.807 | 0.645 |
| CRCR 10 | normal | frozen | 120.7 | 0.991 | 0.973 | 0.942 |
| | prmary | frozen | 129.3 | 0.991 | 0.973 | 0.943 |
| | meta1 | frozen | 140.6 | 0.992 | 0.979 | 0.956 |
| | meta2 | frozen | 89.2 | 0.984 | 0.949 | 0.888 |
